# Hopefulness among individuals living with schizophrenia and their caregivers in Tanzania: an actor-partner interdependence model

**DOI:** 10.1186/s12888-023-04990-8

**Published:** 2023-07-13

**Authors:** Alyssa Martinez, Joy Noel Baumgartner, Sylvia Kaaya, Praxeda Swai, Paul S. Lawala, Beatrice Thedai, Anna Minja, Jennifer Headley, Joseph R. Egger

**Affiliations:** 1 Harvard Medical School, Department of Global Health & Social Medicine, Boston, MA USA; 2 Duke Global Health Institute, Duke University, Durham, NC USA; 3 School of Social Work, University of North Carolina at Chapel Hill, Chapel Hill, NC USA; 4 School of Medicine, Muhimbili University of Health and Allied Sciences, Dar Es Salaam, Tanzania; 5 Mirembe National Mental Health Hospital, Dodoma, Tanzania; 6 Mbeya Zonal Referral Hospital, Mbeya, Tanzania

**Keywords:** Hopefulness, Caregiving, Schizophrenia, Psychotic disorders, Actor-partner interdependence model (APIM)

## Abstract

**Background:**

Hopefulness is a positive orientation or state of mind that can aid in the recovery and treatment of mental illness, as it can have significant impacts on clinical and psychosocial outcomes. As resource-constrained settings work to implement recovery-oriented care, there is a need to better understand hopefulness among people living with schizophrenia (PLWS) and caregivers in their extended family networks. This study seeks to examine the dyadic relationship of hopefulness and its associated correlates among PLWS attending outpatient psychiatric clinics and their caregivers in Tanzania.

**Methods:**

This study utilized baseline and immediate post-intervention data collected as part of a randomized controlled trial testing a culturally tailored model of Family Psychoeducation, KUPAA, in Tanzania. The Herth Hope Index was used to measure hopefulness among PLWS (*n* = 66) and their caregivers (*n* = 66) at baseline and immediate post-intervention. Univariable and multivariable regression models were conducted to determine correlates of hopefulness at baseline, while the Actor-Partner Interdependence Model (APIM) was employed to examine the longitudinal, dyadic relationship of hopefulness among and between PLWS and their caregivers.

**Results:**

Better family functioning was associated with higher levels of hopefulness in PLWS and their caregivers. Lower levels of stigma, lower symptom severity, and lower disability were associated with higher levels of hopefulness in PLWS. For PLWS and their caregivers, actor effects from the APIM model were less than one (PLWS, $\beta=0.261$; caregivers, $\beta=0.318$), indicating stability (within each person) in hopefulness over time. Regarding partner effects, a caregiver’s baseline hopefulness had a positive effect on the hopefulness of their PLWS at follow-up ($\beta=0.100$). This indicates that higher caregiver hope at time 0 is associated with higher levels of hope in PLWS at time 1. Baseline hopefulness levels for PLWS had a negative effect on caregivers’ hopefulness at follow-up ($\beta=-0.106$). This suggests that higher hopefulness among PLWS at baseline is associated with lower levels of hope in caregivers at follow-up.

**Conclusion:**

Hopefulness is important to consider in family or caregiver-based treatments for PLWS because caregiver hopefulness may influence improvements in hopefulness among PLWS over time. Future studies should further explore the longitudinal dyadic relationship of hopefulness for these populations, as hope is a non-pharmacological and modifiable mechanism of change that is underutilized in care and treatment plans for PLWS globally.

**Trial registration:**

Clinical Trials #NCT04013932, July 10, 2019.

**Supplementary Information:**

The online version contains supplementary material available at 10.1186/s12888-023-04990-8.

## Background

Schizophrenia is a severe psychiatric disorder that affects 21 million people worldwide [1]. It can cause significant distress for both the individual living with the disorder and their family members. Schizophrenia is characterized by positive symptoms (e.g., hallucinations, delusions), negative symptoms (e.g., flat emotions, reduced speaking), and/or cognitive symptoms (e.g., attention deficits), and is often chronic in nature with a considerable level of disability [2]. The disorder is treatable, and long-term symptom management and recovery are possible [2, 3].

In low- and middle-income countries like Tanzania and high-income countries like the United States, there are high rates of relapse and rehospitalizations for schizophrenia, highlighting the urgent need for accessible, recovery-oriented care globally [4]. Optional treatment of schizophrenia includes both pharmacological and psychosocial treatment [5]. While psychosocial interventions in combination with medication are more readily available in high-income settings, more resource-limited clinical settings may lack formal treatment options entirely or only offer medication management, which is more often the case for Tanzania [2, 6, 7]. Besides limited resources (providers, hospital beds, etc.), there is no financial security net in Tanzania for individuals living with severe psychiatric disorders [8, 9]. Tanzanian law obliges family members to be the informal caregivers for those with significant psychiatric disabilities, and puts the onus for financial support on the families if assistance is needed [8]. In fact, historically, family members often fulfill roles, such as providing significant social and financial support, for those with more severe psychiatric conditions [10]. Therefore, treatment and recovery may largely depend upon the capabilities of the family caregivers, thus highlighting the importance of better understanding family-involved interventions for schizophrenia.

Existing evidence indicates that families are important for influencing clinical and social outcomes of their relatives living with schizophrenia [11]. For family members to best perform their roles as caregivers and partners in the recovery process, it is important that they too receive support to deal with the associated difficulties and challenges of caregiving [10, 12]. Family interventions that include both caregivers and individuals living with the illness have been found to prevent relapse more effectively than standard care alone [11]. Family interventions, including the evidence-based counseling intervention Family Psychoeducation, have also been found to improve symptoms, and to increase functioning for people living with schizophrenia (PLWS) [13, 14]. Family members who participate in these interventions gain problem-solving and communication skills that may ultimately support their relative’s road to recovery [11, 15].

Hope may be an important construct in recovery-oriented care of schizophrenia because it facilitates agency, self-efficacy, and pathways to healthier lives [3, 16, 17]. A loss of hope is a common experience among individuals living with chronic diseases, including mental health challenges, which negatively impacts the recovery process [3]. An individual’s sense of hope is critical to the personal, social, and clinical recovery processes for severe mental illness as it is often necessary for positive change to occur related to one’s illness. This could have positive implications in low-resource settings where familial care is essential and rehospitalizations are common. While the association between hope and recovery for schizophrenia has been identified, not enough is understood about its mechanisms of action within psychosocial interventions [18].

Hope goes beyond optimism and has been defined several ways across different disciplines [19, 20]. C.R. Snyder defines hope as “the perceived capability to derive pathways to desired goals and motivate oneself via agency thinking to use those pathways” [16]. The creator of the Herth Hope Index, interprets hope as “a multi-dimensional dynamic life force characterized by confident yet uncertain expectation of achieving good, which to the hoping person, is realistically possible and personally significant” [21]. Herth’s definition differs from Snyder’s in that it recognizes one’s interconnectedness with self and others as an important component influencing hope. This suggests that one’s feeling of interconnectedness with their caregiver may influence hope. Hope can also be shaped by cultural context (e.g. role of family, religiosity), making for different experiences and meanings across the globe [19, 22].

Hope can have positive impacts on both patient and caregiver populations. Hopefulness positively influences clinical and psychosocial outcomes, as made evident in several studies with varying chronic indications [19, 20, 23, 24]. Hope specifically enhances coping, particularly for those living with chronic illnesses and their caregivers. A recent study in Dar es Salaam, Tanzania found that hope motivated positive health-seeking behaviors among people living with HIV [23]. A study among older adults in the United States found higher levels of hopefulness to be associated with a reduced risk of all-cause mortality, lower risk of cancer, increased life satisfaction, lower psychological distress, and better social well-being [25]. Studies have additionally identified positive impacts of hope on outcomes related to various mental illnesses like depression, although the research is limited [24, 26, 27].

There is limited quantitative evidence available on the role of hopefulness in improving the mental health of PLWS as well as their caregivers. Most of the existing literature is set in high-income countries, is qualitative, and/or focuses solely on the individuals living with schizophrenia [22, 28–30]. For example, Oles et al. (2015) conducted a quantitative study on the relationship between hope and patient activation among PLWS in the United States and found large, positive concurrent correlations between hope and patient activation [29]. One qualitative study, also set in the U.S., focused on hope in Latinx populations with schizophrenia and their caregivers reported supportive family interactions were “critical for the development and maintenance of hope” [22]. There is some research in Tanzania regarding hope and chronic illnesses, such as HIV/AIDS and cancer, including a recent schizophrenia study that identified an association between high levels of hope and lower caregiver burden [12, 19, 23]. Siril et al., HIV researchers in Tanzania, have called for a better understanding of the role of hopefulness in the treatment and recovery of chronic illnesses [19]. Therefore, there is a need to understand the extent to which hopefulness may be related to recovery in PLWS and their caregivers in Tanzania. Addressing and increasing hope among PLWS and their caregivers may have potential to improve treatment models.

Mental illness does not occur in a vacuum and is instead often experienced within the context of one’s family, which may be even more critical to consider in more collectivist cultures [15]. For family-focused interventions such as Family Psychoeducation, which requires participation of both PLWS and their relatives, examining hopefulness within a dyadic relationship could be particularly illuminating about the role of hope in recovery. The Actor-Partner Interdependence Model (APIM) was developed from the field of psychology and is a widely used analytical model of dyadic relationships [31, 32]. The model accounts for the interdependence within interpersonal relationships, where dyad members may influence each other’s outcomes. Due to the high potential for interdependence of the family caregivers and PLWS in Family Psychoeducation, the APIM model is an appropriate method to examine hopefulness within the caregiving relationship [33].

The aims for this secondary analysis paper were 1) To identify the sociodemographic and illness-related correlates that may be associated with hopefulness separately for PLWS and their caregivers, and 2) To explore the dyadic relationship of hopefulness among PLWS and their caregivers in Tanzania.

## Methods

### Study overview

All data used in this study were collected as part of the pilot individually randomized group treatment trial titled: “Family Psychoeducation for Adults with Psychotic Disorders in Tanzania” (KUPAA), funded by the National Institute of Mental Health (NIMH) [R34MH106663]. KUPAA is a Swahili word meaning ‘to soar’ and it stands for Kuwezeshana kupata uzima which means ‘supporting one another for wholeness’. See Clinicaltrials.gov #NCT04013932 for trial results. Data for the present study included all eligible study participants in both arms of the KUPAA trial.

### Study setting

The KUPAA study was conducted in Dar es Salaam and Mbeya regions, located in the East African country of Tanzania. The first study site was Muhimbili National Hospital (MNH), which is located in the major urban city of Dar es Salaam. MNH is the national referral hospital with a catchment area of about 4 million people. The Department of Psychiatry and Mental Health has both inpatient and outpatient care, with 70 beds in total, usually fully occupied. Psychiatrists, psychiatric nurses, social workers, psychologists, and occupational therapists work together to provide care at this facility.

The study also took place at the Mbeya Zonal Referral Hospital (MZRH), located in the southern highlands zone. MZRH is the only referral facility in the southern part of Tanzania with eight districts and it also acts as a referral facility for neighboring regions. The Psychiatry and Mental Health Unit has 24 beds in total, which are also typically fully occupied. One psychiatrist, along with general practitioners, psychiatric nurses, a psychologist, and social workers provide care at MZRH.

### Participants

A total of 66 dyads composed of individuals living with schizophrenia and their caregivers were included in the study. The study was powered for the primary aim of the clinical trial, which was to assess intervention efficacy, and not for the APIM analysis. All treatment-engaged patient participants had an ICD-10 (International Classification of Disease) diagnosis of either schizophrenia (F20, *n* = 56) or schizoaffective disorder (F25, *n* = 10), were ages 18–50 years at the time of consent, were attending outpatient clinics (prescribed psychotropic medication) had relapsed (defined as inpatient hospitalization for psychiatric reasons) in the past year. Patients were recruited on the day that they arrived for their outpatient appointments while they were in the waiting areas and in consultation with clinicians who referred clients who met study criteria. Caregiver participants were identified by individuals living with the psychotic disorder and were at least 18 years old. Caregivers could have been a family member, spouse, friend, etc. of the PLWS, and they were often the same person who accompanied the patients to their outpatient appointments, a practice that is very common in this setting, although patients were allowed to select anyone.

### Procedures

Baseline data collection took place in September and October of 2019, the intervention was delivered from November 2019 to February 2020, and immediate post-intervention follow-up data collection occurred from March through June of 2020. Written informed consent was obtained from all participants after being screened for study eligibility. Individuals living with schizophrenia were required to be stable at the time of consent, which was determined by the study psychiatrists. To ensure that these individuals were able to give adequate informed consent, the research team revisited the consent form with them prior to the follow-up interview. All participants were compensated 7,500 Tsh (~ $3.50 USD) at each interview for costs related to study attendance.

Study visits and data collection occurred in office facilities within MNH and MZRH. Research assistants administered all patient interviews with self-reported assessment measures, including sociodemographic information, except for the clinician-rated measure, the Positive and Negative Syndrome Scale (PANSS) [34], in a single session lasting around 2 h. The PANSS was administered in a separate interview by a study psychiatrist or clinical psychologist. Caregiver interviews were brief and administered separately to ensure confidentiality. Interviews were carried out in Kiswahili, the official language in Tanzania. Data were collected electronically on tablets using REDCap software.

### Measures

This study includes measures for PLWS and their caregivers. The World Health Organization’s four-step translation and cultural validation process, namely, forward-translation, back-translation, pre-testing, and finalization with expert consensus, was implemented for all scales in the study [35].

Hopefulness, our dependent variable in the regression and APIM analyses, was measured using the Herth Hope Index (HHI) [21]. This was completed by all PLWS and their caregivers about their own hopefulness. The HHI was administered at baseline (pre-intervention) and post-intervention which allowed for an estimation of changes in hope over time. The HHI contains three factors including temporality and future (*n* = 4 items), positive readiness and expectancy(*n* = 4 items), and interconnectedness (*n* = 4 items) [21]. Factor 1 attempts to measure the perception that a positive outcome is possible in the future, while factor 2 attempts to measure the feeling of confidence to initiate action plans. Factor 3 assesses the recognition of interdependence between self and others [21]. Scores range from 12–48, with a higher HHI total indicating a higher level of hopefulness. Questions are scored using a four-point Likert scale ranging from 1 (strongly disagree) to 4 (strongly agree). Scoring entails an unweighted summing of item points for subscales and the total scale. Reverse scoring is used for items #3 and #6 as they are worded negatively. The internal consistency of the scale was very good in this study. Baseline Cronbach’s alpha scores for patients and their caregivers were 0.89 and 0.92, respectively.

Religiosity was measured for all participants using the Duke University Religion Index (DUREL) [36]. DUREL is a 5-item scale that measures religious involvement. The scale assesses three major dimensions of religiosity including organizational religious activity (ORA), non-organizational religious activity (NORA), and intrinsic religiosity (IR). The DUREL instrument is designed so that each dimension is to be measured and analyzed separately. This study utilized the 3-item IR subscale for analysis with three items, which refers to one’s degree of personal religious commitment. DUREL-IR scores range from 3 to 15, with higher scores indicating higher levels of religious commitment. The Cronbach’s alpha for the IR subscale was 0.78 in this study, indicating acceptable reliability.

The Internalized Stigma of Mental Illness (ISMI) scale assesses experiences with stigma in participants living with schizophrenia [37]. The scale consists of 29 items scored on a 4-point Likert scale. The instrument includes statements such as “I feel out of place in the world because I have mental illness.” The ISMI measures five subscales of stigma including alienation (*n* = 6 items), stereotype endorsement (*n* = 7 items), discrimination experience (*n* = 5 items), social withdrawal (*n* = 6 items), and stigma resistance (*n* = 5 items). Higher scores reflect higher levels of internalized stigma. Internal consistency of the scale was good in this study with a Cronbach’s alpha of 0.92.

The severity of symptoms experienced by PLWS was measured using the clinician-rated Positive and Negative Syndrome Scale (PANSS) [34]. The PANSS is a 30-item structured interview comprised of 7 positive and 7 negative symptom items, in addition to 16 general psychopathology items. Items are ranked on a 7-point Likert scale, with symptom subscale scores ranging from 7 to 49 and general psychopathology scores ranging from 16 to 112. Higher scores indicate a higher level of symptom severity. The Cronbach’s alpha was 0.91, indicating high internal consistency.

Family functioning was reported by all participants using the 15-item version of the Systemic Clinical Outcome and Routine Evaluation (SCORE-15) [38, 39]. The SCORE-15 is a questionnaire that can be used to measure therapeutic changes in functioning for those engaged in family or couples therapy. The instrument consists of three dimensions including strengths and adaptability (5 items), overwhelmed by difficulties (5 items), and disrupted communication (5 items). Items are rated on a 5-point Likert scale and total scores range from 15 to 75, with lower scores indicating better family functioning. The Cronbach’s alpha was 0.82 for both PLWS and caregivers, indicating good reliability.

The level of disability in PLWS was assessed using the World Health Organization Disability Assessment Schedule-Second Version (WHODAS 2.0) [40]. The WHODAS 2.0 is constructed of 36-items in a Likert format that are divided into the domains of understanding and communicating, getting around, self-care, getting along with people, life activities, and participation in society. The complex scoring approach was used, with total scores ranging from 0 (no disability) to 100 (full disability). The Cronbach’s alpha for PLWS was 0.96, indicating high reliability.

Burden experienced by caregiver participants was measured utilizing the Burden Assessment Scale (BAS) [41]. The BAS consists of 19-items measuring objective and subjective consequences of providing care to a relative with mental illness. Items are rated on a 4-point Likert scale and total scores range from 19 to 76, with higher scores indicating higher levels of caregiver burden. The BAS had very high internal consistency in this study (Cronbach’s alpha = 0.95).

### Data management and analysis

Descriptive statistics were generated for all study participants. Continuous variables were summarized by their mean and standard deviation (SD), while categorical variables were summarized as counts and percentages. Internal consistency of the psychometric instruments was measured by calculating the unstandardized Cronbach’s alpha using baseline data for caregivers and PLWS as appropriate.

Univariable and multivariable linear regression modeling was performed to identify potential correlates of hopefulness (HHI total, dependent variable)) separately in individuals living with schizophrenia and their caregivers using all variables presented in Tables 1 and 2. The regression analysis for PLWS included the full sample (*n* = 66) and a nearly full sample (*n* = 65) for caregivers, as one caregiver had incomplete data. Mean imputations were performed to handle missing values for the HHI, WHODAS, and SCORE-15 instruments. Model fit of continuous dependent variables was assessed using R^2^, adjusted R^2^, F-statistics, and scatter plots of residuals. Linear and non-linear associations between dependent and independent variables were considered. All psychometric measures were fit as continuous variables. Bivariate associations were summarized by their mean difference and 95% confidence interval. Due to the small sample size and lack of a strong a priori understanding of the relationships, we chose to fit minimally adjusted models for both participant groups. For PLWS, models were adjusted for sex, length of illness, and symptom severity. Age was not included as a covariate as it was highly correlated with length of illness for patients. For caregivers, models were adjusted for sex and age.

**Table 1:** Characteristics of individuals living with schizophrenia stratified by sex

	Total (*N* = 66)	Men (*N* = 44)	Women (*N* = 22)
Age, in years			
Mean (SD)	33.0 (8.2)	32.6 (8.2)	33.9 (8.4)
Min, Max	18, 50	18, 49	21, 50
Age Categorized			
< = 24	10 (15.2%)	7 (15.9%)	3 (13.6%)
25–34	27 (40.9%)	20 (45.5%)	7 (31.8%)
35–50	29 (43.9%)	17 (38.6%)	12 (54.6%)
Relationship Status			
Partnered, living together	10 (15.2%)	7 (15.9%)	3 (13.6%)
Partnered, not living together	12 (18.2%)	4 (9.1%)	8 (36.4%)
Single, not partnered	44 (66.6%)	33 (75.0%)	11 (50.0%)
Educational Level			
Primary or Less	25 (37.9%)	15 (34.1%)	10 (45.5%)
Secondary or Higher	41 (62.1%)	29 (65.9%)	12 (54.5%)
Worked in the Past 3 Months			
No	28 (42.4%)	16 (36.4%)	12 (54.5%)
Yes	38 (57.6%)	28 (63.6%)	10 (45.5%)
Religion			
Muslim	18 (27.3%)	13 (29.5%)	5 (22.7%)
Catholic	9 (13.6%)	5 (11.4%)	4 (18.2%)
Christian/Protestant	38 (57.6%)	25 (56.8%)	13 (59.1%)
Other	1 (1.5%)	1 (2.3%)	0 (0.0%)
Length of illness, in years			
Mean (SD)	9.1 (8.1)	9.0 (7.5)	9.4 (9.3)
Min, Max	0, 29	0, 29	0, 26
Hope (HHI)			
Mean (SD)	34.0 (7.0)	35.1 (6.6)	31.7 (7.3)
Min, Max	20, 48	24, 48	20, 46
Symptom Severity (PANSS Total)			
Mean (SD)	45.9 (14.5)	48.0 (16.4)	41.7 (8.9)
Min, Max	30, 103	30, 103	30, 67
Disability (WHODAS 2.0, Complex)			
Mean (SD)	37.5 (20.6)	37.2 (20.5)	38.1 (21.3)
Min, Max	0, 83.7	0, 82.6	4.3, 83.7
Religiosity (DUREL)			
Mean (SD)	13.1 (1.6)	13.00 (1.6)	13.3 (1.8)
Min, Max	9,15	9,15	9,15
Internalized Stigma (ISMI)			
Mean (SD)	2.4 (0.5)	2.3 (0.50)	2.43 (0.5)
Min, Max	1.2,3.7	1.2,3.7	1.6,3.3
Family Functioning (SCORE-15)			
Mean (SD)	2.6 (0.6)	2.6 (0.5)	2.7 (0.7)
Min, Max	1.7,4.5	1.7,3.8	1.6,4.5

*HHI* Herth Hope Index (Higher score means more hopeful), *PANSS* Positive and Negative Syndrome Scale (Higher score means more symptom severity), *WHODAS* World Health Organization Disability Assessment Schedule (Higher score means more disability), *DUREL* Duke University Religion Index (Higher score means higher religiosity), *ISMI* Internalized Stigma of Mental Illness (Higher score means more internalized stigma), SCORE-15, Family Functioning (Higher score means worse family functioning)

**Table 2:** Characteristics of caregivers stratified by sex

	Total (*N* = 66)	Men (*N* = 23)	Women (*N* = 43)
Age, in years			
Mean (SD)	48.8 (13.1)	47.0 (15.3)	49.7 (11.9)
Min, Max	21, 72	21, 72	25, 70
Age Categorized			
< = 24	1 (1.5%)	1 (4.4%)	0 (0.0%)
25–49	32 (48.5%)	13 (56.5%)	19 (44.2%)
50–74	33 (50.0%)	9 (39.1%)	24 (55.8%)
Relationship Status			
Partnered, living together	34 (51.5%)	14 (60.9%)	20 (46.5%)
Partnered, not living together	9 (13.6%)	4 (17.4%)	5 (11.6%)
Single, not partnered	23 (34.9%)	5 (21.7%)	18 (41.9%)
Educational Level			
Primary or Less	42 (63.6%)	14 (60.9%)	28 (65.1%)
Secondary or Higher	24 (36.4%)	9 (39.1%)	15 (34.9%)
Worked in the Past 3 Months			
No	31 (47.0%)	11 (47.8%)	20 (46.5%)
Yes	35 (53.0%)	12 (52.2%)	23 (53.5%)
Religion			
Muslim	15 (23.1%)	4 (18.2%)	11 (25.6%)
Catholic	11 (16.9%)	2 (9.1%)	9 (20.9%)
Christian/Protestant	38 (58.5%)	16 (72.7%)	22 (51.2%)
Other	1 (1.5%)	0 (0.0%)	1 (2.3%)
Living with the participant			
No	10 (15.2%)	4 (17.4%)	6 (14.0%)
Yes	56 (84.8%)	19 (82.6%)	37 (86.0%)
Relationship to Individual with Schizophrenia			
Partner	7 (10.6%)	2 (8.7%)	5 (11.6%)
Child	2 (3.0%)	1 (4.3%)	1 (2.3%)
Parent	32 (48.5%)	7 (30.4%)	25 (58.1%)
Sibling	9 (13.6%)	4 (17.4%)	5 (11.6%)
Other relatives	15 (22.7%)	8 (34.8%)	7 (16.3%)
Friend	1 (1.5%)	1 (4.4%)	0 (0.0%)
Hope (HHI)			
Mean (SD)	38.7 (6.7)	39.1 (6.6)	38.6 (6.8)
Min, Max	14, 48	25, 48	14, 48
Caregiver Burden (BAS)			
Mean (SD)	45.8 (15.7)	43.2 (17.4)	47.1 (14.8)
Min, Max	19, 75	19, 75	22, 73
Family Functioning (SCORE-15)			
Mean (SD)	2.4 (0.6)	2.3 (0.6)	2.5 (0.6)
Min, Max	1.1,3.6	1.3,3.5	1.1,3.6

*HHI* Herth Hope Index (Higher score means higher hopefulness), *BAS* Burden Assessment Scale (Higher score means more burden), SCORE-15, Family Functioning (Higher score means worse family functioning)

**Fig. 1:**
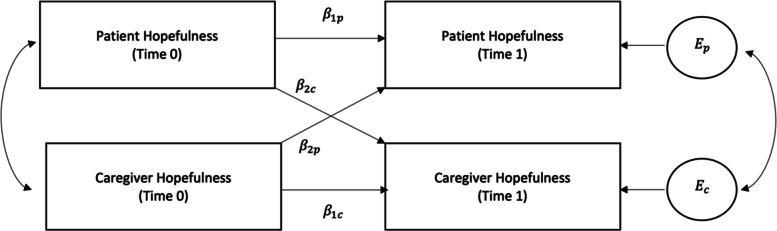
Actor-partner interdependence model of hopefulness. Time 0 is baseline data collection and time 1 is data collection immediately post-intervention

The analytic technique called Actor-Partner Interdependence Model (APIM) was employed to account for the non-independence of the caregiver and PLWS dyads. Complete dyad data for 65 pairs and partial dyad data for 1 pair was used in the APIM analysis. Figure 1 depicts the repeated measures APIM framework of a caregiver-patient dyad in which there is one variable at two different timepoints from each dyad member: hopefulness at baseline and 3 month follow-up [42]. The model estimates individual-level effects of a predictor, otherwise known as actor effects (e.g., how a PLWS’s level of hope at time 0 affects his or her own level of hope at time 1). APIM also simultaneously estimates partner effects (e.g., how a caregiver’s level of hope at time 0 impacts the PLWS’s level of hope at time 1), which are the effects of a predictor from the dyad partner on the paired individual’s outcome [31, 32]. More specifically, the model estimates the mean HHI total as a function of actor and partner effects. In this study, the dyads were treated as distinguishable based off on the theoretical distinction between roles (caregiver vs. PLWS). The two-intercept approach in multilevel modeling was used to obtain the actor-partner effects per level of the distinguishing variable (e.g., dyadic role). The original dataset was restructured into a pairwise organization with each dyad participant occupying a separate record. Two dummy variables for the distinguishing variable were coded and included in the model separately and as interaction terms (caregiver = 1 if caregiver, caregiver = 0 if patient; and patient = 1 if patient, patient = 0 if caregiver). Four variables were then created using dummy indicator variable coding and included in the model:

$${\beta }_{1p}=actor\ effect\ among\ patients$$

$${\beta }_{1c}=actor\ effect\ among\ caregivers$$

$${\beta }_{2c}=partner\ effect\ among\ caregivers$$

$${\beta }_{2p}=partner\ effect\ among\ patients$$

The residual errors on hopefulness for caregivers and patients are represented by ${\mathrm{E}}_{\mathrm{c}}$ and ${\mathrm{E}}_{\mathrm{p}}$ in Fig. 1. Beta coefficients were estimated with a linear mixed model using baseline HHI totals for each dyad member as appropriate and assumed an exchangeable correlation matrix on the residuals. Models were adjusted for study arm (design variable). STATA 17.0 software was used to perform all statistical analyses [43]. Due to the exploratory nature of the study, no null hypothesis significance testing was performed.

## Results

### Participant characteristics

Table 1 shows the sociodemographic and clinical characteristics of the PLWS stratified by sex. The average age of PLWS was 33 years (SD = 8.2) and the majority were men (66.7%). Most PLWS reported being single (66.6%) and having worked in the last three months (57.6%). The majority completed secondary school or obtained a higher education (62.1%). The average length of illness was 9 years. The mean hopefulness score (HHI) was 35.1 (SD = 6.6) in men and 31.7 (SD = 7.3) in women. The mean symptom severity score (PANSS total) was 45.9 (SD = 14.5) and the mean disability score (WHODAS 2.0) was 37.5 (SD = 20.6).

Table 2 shows the sociodemographic characteristics of caregivers stratified by sex. The average age of caregivers was 48.8 years (SD = 13.2), and the majority were women (65.2%). Most caregivers reported being partnered and living together (51.5%). More than half of the caregivers had worked in the past three months (53.0%) and the majority had completed primary education (63.6%). Nearly all caregivers reported living with the affected individual (84.8%). Most caregivers were parents to the affected individual (48.5%). The mean hopefulness score (HHI) was 38.7 (SD = 6.7). The average burden score (BAS) was 43.2 (SD = 17.4) for men and 47.1 (SD = 14.8) for women.

### Correlates of hopefulness

Supplemental Table 1 provides baseline and follow-up HHI means by item for both PLWS and caregivers. Table 3 shows crude and adjusted results of the linear regression analysis estimating the associations between HHI total and characteristics of PLWS. HHI total was approximately linearly associated with the psychometric measures. Higher (better) hopefulness (HHI) totals among PLWS were associated with lower levels of stigma (ISMI) reported by PLWS, better family functioning (SCORE-15) reported by PLWS, and less disability (WHODAS 2.0) experienced by PLWS. Little difference in crude and adjusted models was observed on adjustment for sex, length of illness, and symptom severity (PANSS).

**Table 3:** Results of univariable and multivariable linear regression models estimating mean hopefulness for PLWS

Independent variable	*N*	Crude model Mean change, HHI (95% CI)	Adjusted model Mean change, HHI (95% CI)
Internalized Stigma (ISMI)	66	-9.96 (-12.47, -7.45)	-8.86 (-11.37, -6.36)
Symptom Severity (PANSS)	66	-0.15 (-0.26, -0.04)	-0.17 (-0.29, -0.06)
Family Functioning (SCORE-15)	66	-6.30 (-8.85, -3.76)	-5.39 (-7.94, -2.84)
Disability (WHODAS 2.0)	66	-0.23 (-0.30, -0.17)	-0.24 (-0.31, -0.16)

Table 4 shows the crude and adjusted results of the linear regression analysis estimating the associations between caregiver hopefulness (HHI) totals and caregiver characteristics. Caregivers living in the same household as their relative living with schizophrenia had a higher average hope total than those who were not cohabitating (mean diff: 2.15; 95% CI: -2.43, 6.74). Mean HHI totals were approximately linearly associated with the psychometric measures of caregiver reports of burden and family functioning. Higher (better) caregiver HHI totals were associated with lower levels of caregiver burden (BAS) and better family functioning (SCORE-15). On adjustment for age and sex, little differences from the crude models were observed.

**Table 4:** Results of univariable and multivariable linear regression models estimating mean hopefulness for caregivers

Independent variable	*N*	Crude model Mean change, HHI (95% CI)	Adjusted model Mean change, HHI (95% CI)
**Living with Patient**			
No (REF)	10	36.90 (32.68, 41.12)	36.25 (32.11, 40.39)
Yes	55	39.05 (37.26, 40.85)	39.17 (37.42, 40.92)
*Difference*	65	2.15 (-2.43, 6.74)	2.92 (-1.59, 7.43)
**Family Functioning (SCORE-15)**	65	-4.39 (-7.00, -1.78)	-4.91 (-7.44, -2.37)
**Caregiver Burden (BAS)**	65	-0.27 (-0.35, -0.19)	-0.27 (-0.35, -0.19)

### APIM analysis

**Fig. 2:**
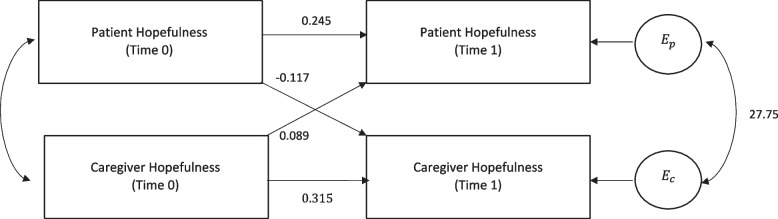
Results of APIM analysis. Time 0 is baseline data collection and time 1 is data collection immediately post-intervention

The results of the APIM analysis are depicted in Fig. 2. For both people living with schizophrenia and their caregivers, positive actor effects less than one were observed, indicating stability of the hopefulness measure over time within individuals. (For PLWS, $\upbeta =0.245(95\mathrm{\% CI}:0.039, 0.451)$; for caregivers, $\upbeta =0.315 (95\mathrm{\% CI}:0.098, 0.532)$). With regard to partner effects, caregivers’ baseline hopefulness had a positive effect on PLWS’ hopefulness at follow-up ($\upbeta =0.089;95\mathrm{\% CI}: -0.131, 0.308$). Baseline hopefulness levels for people living with schizophrenia had a negative effect on caregiver hopefulness at follow-up ($\upbeta = -0.117;95\mathrm{\% CI}: -0.321, 0.088$).

In an APIM model, the joint effect, or the additive effect of both actor and partner baseline HHI score on follow-up actor HHI score, differs depending on the level and direction of baseline scores. The mean HHI scores at baseline and follow-up of both caregivers and PLWS are displayed in Table 5.

**Table 5:** Mean total Hopefulness score, by Time, in PLWS & Caregivers

Timepoint	Mean HHI Total (SD)	
	*PLWS*	*Caregivers*
*Baseline*	33.98 (6.98)	38.72 (6.67)
*Follow-Up*	37.30 (6.73)	41.00 (5.97)

**Fig. 3:**
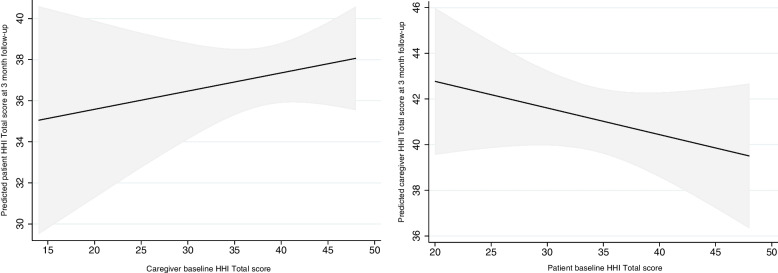
Partner effects for caregivers and patients. Predicted patient HHI Total score at post-intervention follow-up by changes in caregiver baseline score, holding patient baseline HHI Total score at its observed mean (left panel); and predicted caregiver HHI Total score at post-intervention follow-up by changes in patient baseline score, holding caregiver baseline HHI Total score at its observed mean (right panel)

Table 6 and Fig. 3 illustrate these joint effects from our model for a hypothetical patient in the intervention (KUPAA) arm of the study. Each row of Table 6 depicts different baseline hopefulness (HHI) scores for a PLWS patient (actor) and their caregiver; specifically, assigning baseline score for each participant at the mean value, as well as 1 standard deviation below and above this mean. We can see that within levels of the actor’s baseline score, as their caregiver’s baseline HHI score increases from 1 SD below the mean to 1 SD above the mean, the model predicts a 1.24 unit increase in follow-up PLWS HHI score. This increase in score is the same, regardless of baseline actor HHI score and represents the main caregiver effect from the model. However, when looking at the joint effects of actor and caregiver, we can see that the change in the actor’s HHI score from baseline to follow-up depends on the level of the baseline scores. For example, if we hold the actor’s baseline HHI score at 1 SD above the mean, we see only very little change in follow-up actor’s HHI score, regardless of caregiver’s baseline score (range: -1.5% to 1.6%); however, if we hold the actor’s baseline HHI score at 1 SD below the mean, we see a greater increase in follow-up actor HHI score (range: 36.9% to 41.5%). This suggests that those PLWS participants with lower baseline HHI scores saw a greater improvement in follow-up HHI score than PLWS participants with higher baseline HHI score, and this level of improvement was greater still if their caregiver had a higher baseline HHI total score.

**Table 6:** Model predicted follow-up HHI Total score values for hypothetical values of baseline actor and partner HHI Total scores for a PLWS in the intervention (KUPAA) trial arm

**PLWS (Actor)**	**Caregiver (Partner)**	**Predicted F/Up HHI Total**	**HHI difference**	**Absolute change from baseline actor value**	**Relative change from baseline actor value**
**HHI Total at Baseline**	**HHI Total at Baseline**				
Mean (34)	1 SD—Mean (32)	38.69	–	4.69	13.8%
Mean (34)	Mean (39)	39.31	0.62	5.31	15.6%
Mean (34)	1 SD + Mean (46)	39.93	1.24	5.93	17.4%
1 SD + Mean (41)	1 SD – Mean (32)	40.41	–	-0.59	-1.5%
1 SD + Mean (41)	Mean (39)	41.03	0.62	0.03	0.1%
1 SD + Mean (41)	1 SD + Mean (46)	41.65	1.24	0.65	1.6%
1 SD – Mean (27)	1 SD – Mean (32)	36.98	–	9.98	36.9%
1 SD – Mean (27)	Mean (39)	37.60	0.62	10.60	39.2%
1 SD – Mean (27)	1 SD + Mean (46)	38.22	1.24	11.22	41.5%

## Discussion

The current study examined the independent and dyadic associations between hopefulness and selected predictors within the PLWS-caregiver relationship. Hopefulness in our study population exhibits interdependence meaning dyad partners influence one another’s level of hope over time. To the best of our knowledge, there is no current literature on the interdependence of hopefulness among PLWS and their caregivers for comparison. Family functioning was found to be an important correlate of hopefulness among both participant groups. Better family functioning was associated with higher levels of hopefulness, suggesting that the maintenance of a healthy relationship with one’s dyadic partner is essential. This finding speaks to the interdependent nature of hopefulness among this population and suggests that facilitating healthy relationship dynamics could promote positive outcomes in both PLWS and caregivers.

Among PLWS, we found various psychosocial and clinical factors to be important correlates of hopefulness. Precisely, lower levels of internalized stigma, symptom severity, and disability were associated with higher levels of hopefulness. This aligns with findings from the limited studies that have explored correlates of hopefulness among PLWS [44–46]. These findings are correlations and do not indicate causation. Therefore, hope may also be contributing to reduced stigma, symptom severity, and disability.

A positive partner effect was identified among PLWS. This indicates that higher caregiver hopefulness is associated with higher patient hopefulness at follow-up. Therefore, caregivers may play a significant role in influencing hopefulness levels among PLWS. Facilitating hopefulness in caregivers may ultimately be important for improving psychosocial and clinical outcomes in PLWS. A negative partner effect among caregivers was identified, indicating that higher hopefulness among PLWS is associated with lower caregiver hopefulness at follow-up. This finding is only somewhat unexpected. There is literature to indicate that PLWS who have low insight (illness awareness) have more difficulties in the recovery process which could include goal setting alignment with clinicians and caregivers who are more or less hopeful about what is possible for the future given symptomology and functioning [47, 48]. Therefore, if PLWS have misguided or overly optimistic hopefulness about levels or types of recovery, caregivers could become less hopeful over time. Future studies should seek to confirm these exploratory findings.

This study has several limitations, including its small sample size of dyads (*n* = 66). As the present study was a secondary analysis of data captured in the KUPAA RCT, it was not powered for the APIM analysis. Results should be interpreted in an exploratory manner. Future studies with larger samples of participant dyads, and longer follow-up should be able to estimate actor and partner effects more precisely, as well as investigate how the dyadic relationship of hopefulness changes over time.

Second, hopefulness is a latent construct that is subject to measurement error. The HHI instrument has not been validated in Tanzania and may not have accurately captured the experience of hopefulness in our study population despite instrument translation and adaptation. It is worth noting that the Herth Hope scale was found to be highly correlated in our data with a second local measure of hopefulness, the Helen Siril Hope scale, which was originally developed for HIV populations (ρ_pearson_ = 0.88; ρ_spearman_ = 0.90), lending some confidence to indicate local face validity [19]. Regardless, future research is needed to validate the HHI tool more thoroughly among this population in Tanzania.

Lastly, the study sample may be not representative of the larger population of PLWS and their caregivers in Tanzania. Participants were eligible to participate if they were receiving outpatient services at either of the study sites. This criterion likely excluded several affected individuals due to the logistical and financial challenges associated with accessing care in Tanzania. Additionally, symptoms among PLWS had to be stable at the time of informed consent which likely led to the exclusion of affected individuals who were experiencing an acute episode of their illness at the time of study recruitment. As many of the PLWS in our study are reliant on their relatives for organizing their treatment and appointments, our study may exclude less involved caregivers. Consequently, hopefulness levels may be higher in the present study than in the general population of PLWS in the areas studied.

Tanzania’s Disabilities Act of 2010, which legally requires informal family caregivers to take financial and social responsibility for individuals with disabling levels of mental illness, could be improved upon with a government-backed statutory financial safety net. While caregivers are needed for a range of psychosocial supports, removing the additional financial burden could help them better carry out their roles effectively while remaining hopeful. Additionally, caregivers should be included in clinic and community-based interventions targeted at fostering hopefulness alongside patients. Similar to the intervention implemented by Chan et al. in persons recovering from cancer, a clinic-based hope intervention may consist of therapy on topics including goal setting, identification of pathways to achieve goals, and positive self-talk [49]. Laranjeira and Querido suggest hope-inspiring competence should be considered a key skill for mental health professionals and this skill would fit well with the family psychoeducation model [50]. This study further illustrates the inter-relatedness of patients and caregivers, such that healthcare providers and health systems should consider the added value of including families in clinical care, including a focus on fostering hopefulness.

To the best of our knowledge, this study is the first to examine the longitudinal, dyadic relationship of hopefulness among PLWS and their caregivers in resource limited settings, such as Tanzania. As indicated in other studies conducted in Tanzania, family structures, roles, and responsibilities are both traditional and evolving with rapid social change (e.g. deferential respect for parents/elders who are often caregivers, alongside a potential lessening of extended family support for caregiving, moving towards more nuclear family structures, particularly in urban areas [51–53]. In order to gain a wider understanding of the issue at hand, future research should be more inclusive of participants’ illness severity and accessibility of mental health services so as to have a more representative study population. Future studies may also consider including additional predictors of hopefulness using the APIM model.

Qualitative research may be particularly beneficial to conduct on this topic, as hopefulness is a multifaceted construct that is influenced by culture and context. Qualitative research should be conducted among both PLWS and their caregivers in order to further reveal the complexities of hopefulness as experienced in the family context.

## Conclusion

The results of our study suggest that hopefulness may be important to consider in PLWS treatment regimens as caregiver hopefulness is associated with improvements in PLWS hopefulness over time. Dyads of this nature are complex, and members are continuously influencing each other’s outcomes. Neither schizophrenia nor hopefulness are experienced in a vacuum. Therefore, caregiver mental health and well-being are absolutely critical to consider when working to promote recovery in individuals living with schizophrenia. Hope is a powerful non-pharmacological tool that is underutilized in both high-resource and resource-constrained settings.

## Supplementary Information

**Additional file 1.** **Supplemental Table 1. **HHI Item Scores by Time for PLWS & Caregivers. 

## Data Availability

The dataset generated and analyzed during the current study is not publicly available per IRB requirements, but it is available from the senior author (JRE) on reasonable request, with permission from study PIs, and with IRB approval for secondary analyses to maintain confidentiality.

## Abbreviations

KUPAA: Kuwezeshana kupata uzima (Swahili for, 'supporting one another for wholeness')
PLWS: People living with schizophrenia
APIM: Actor-Partner interdependence model
MUHAS: Muhimbili University of health and allied sciences
NIMH: National Institute of mental health
MNH: Muhimbili National Hospital
MZRH: Mbeya zonal referral hospital
ICD-10: International classification of disease, 10th edition
HHI: Herth hope index
DUREL: Duke University religion index
ISMI: Internalized stigma of mental illness
PANSS: Positive and negative syndrome Scale
SCORE-15: Systemic clinical outcome and routine evaluation
WHODAS 2.0: World Health Organization disability assessment schedule- second version
BAS: Burden assessment scale

## References

[1] Charlson FJ, Ferrari AJ, Santomauro DF, Diminic S, Stockings E, Scott JG, et al. Global epidemiology and burden of schizophrenia: findings from the global burden of disease study 2016. Schizophr Bull. 2018;44(6):1195–203. 29762765 10.1093/schbul/sby058 PMC6192504

[2] World Health Organization. Schizophrenia. World Health Organization. 2019. Cited 2021 Nov 7. Available from: https://www.who.int/news-room/fact-sheets/detail/schizophrenia.

[3] Noordsy D, Torrey W, Mueser K, Mead S, O’Keefe C, Fox L. Recovery from severe mental illness: an intrapersonal and functional outcome definition. Int Rev Psychiatry. 2002;14(4):318–26.

[4] Hudson CG. Five-year rehospitalization experience of a state-wide cohort of persons with schizophrenia. Soc Psychiatry Psychiatr Epidemiol. 2019;54(7):861–70. 30603806 10.1007/s00127-018-1650-7

[5] NIMH. NIMH » Schizophrenia. NIMH. Cited 2022 Jan 27. Available from: https://www-nimh-nih-gov.proxy.lib.duke.edu/health/topics/schizophrenia.

[6] Aloyce F. Factorsinfluencing antipsychotic medication non-adherence amongst persons withpsychotic disorders at the Muhimbili National Hospital, Dar es Salaam,Tanzania. MMed (Psychiatry) Dissertation. [Doctoral dissertation]. University of Dar es Salaam; 2007.

[7] Baumgartner JN. Measuringdisability and social integration among adults with psychotic disorders in Dares Salaam, Tanzania. [Doctoral dissertation]. UNC Chapel Hill; 2004.

[8] Tanzania Government. The Persons with Disabilities Act 2010 . 2010. Cited 2021 Dec 6. Available from: https://www.tanzania.go.tz/egov_uploads/documents/The_Persons_with_Disabilities_Act,_2010_(Act_No_sw.pdf.

[9] de Jesus MJ, Razzouk D, Thara R, Eaton J, Thornicroft G. Packages of care for schizophrenia in low- and middle-income countries. PLoS Med. 2009;6(10):e1000165. 19841735 10.1371/journal.pmed.1000165 PMC2758997

[10] Caqueo-Urízar A, Rus-Calafell M, Craig TKJ, Irarrazaval M, Urzúa A, Boyer L, et al. Schizophrenia: impact on family dynamics. Curr Psychiatry Rep. 2017;19(1):2. 28097634 10.1007/s11920-017-0756-z

[11] Bighelli I, Rodolico A, García-Mieres H, Pitschel-Walz G, Hansen W-P, Schneider-Thoma J, et al. Psychosocial and psychological interventions for relapse prevention in schizophrenia: a systematic review and network meta-analysis. Lancet Psychiatry. 2021;8(11):969–80. 34653393 10.1016/S2215-0366(21)00243-1

[12] Clari R, Headley J, Egger J, Swai P, Lawala P, Minja A, et al. Perceived burden and family functioning among informal caregivers of individuals living with schizophrenia in Tanzania: a cross-sectional study. BMC Psychiatry. 2022;22(1):10. 34983438 10.1186/s12888-021-03560-0 PMC8728903

[13] Xia J, Merinder LB, Belgamwar MR. Psychoeducation for schizophrenia. Cochrane Database Syst Rev. 2011;6:CD002831. 10.1002/14651858.CD002831.pub2 PMC4170907 21678337

[14] Dixon LB, Lehman AF. Family interventions for schizophrenia. Schizophr Bull. 1995;21(4):631–43. 8749890 10.1093/schbul/21.4.631

[15] Hinton L, Kohrt BA, Kleinman A. Engaging families to advance global mental health intervention research. Lancet Psychiatry. 2019;6(5):365–7. 30954478 10.1016/S2215-0366(19)30134-8 PMC6848916

[16] Snyder CR. Hope theory: rainbows in the mind. Psychol Inq. 2002;13(4):249–75.

[17] Schrank B, Slade M. Recovery in psychiatry. Psychiatr Bull. 2007;31(9):321–5.

[18] Kylmä J, Juvakka T, Nikkonen M, Korhonen T, Isohanni M. Hope and schizophrenia: an integrative review. J Psychiatr Ment Health Nurs. 2006;13(6):651–64. 17087667 10.1111/j.1365-2850.2006.01012.x

[19] Siril H, Smith Fawzi MC, Todd J, Somba M, Kaale A, Minja A, et al. The value of hope: development and validation of a contextual measure of hope among people living with HIV in urban Tanzania a mixed methods exploratory sequential study. BMC Psychol. 2020;8(1):5. 31996246 10.1186/s40359-020-0376-y PMC6988347

[20] Schiavon CC, Marchetti E, Gurgel LG, Busnello FM, Reppold CT. Optimism and hope in chronic disease: a systematic review. Front Psychol. 2016;7:2022. 28101071 10.3389/fpsyg.2016.02022 PMC5209342

[21] Herth K. Abbreviated instrument to measure hope: development and psychometric evaluation. J Adv Nurs. 1992;17(10):1251–9. 1430629 10.1111/j.1365-2648.1992.tb01843.x

[22] Hernandez M, Barrio C, Gaona L, Helu-Brown P, Hai A, Lim C. Hope and schizophrenia in the latino family context. Community Ment Health J. 2019;55(1):42–50. 30506465 10.1007/s10597-018-0354-5 PMC6629030

[23] Siril H, Fawzi MCS, Todd J, Wyatt M, Kilewo J, Ware N, et al. Hopefulness fosters affective and cognitive constructs for actions to cope and enhance quality of life among people living with HIV in Dar Es Salaam, Tanzania. J Int Assoc Provid AIDS Care. 2017;16(2):140–8. 24963087 10.1177/2325957414539195 PMC7903482

[24] Mansano-Schlosser TC, Ceolim MF, Valerio TD. Poor sleep quality, depression and hope before breast cancer surgery. Appl Nurs Res. 2017;34:7–11. 28342628 10.1016/j.apnr.2016.11.010

[25] Long KNG, Kim ES, Chen Y, Wilson MF, Worthington EL Jr, VanderWeele TJ. The role of Hope in subsequent health and well-being for older adults: an outcome-wide longitudinal approach. Glob Epidemiol. 2020;2: 100018.

[26] Hsu S-Y, Huang H-S. improving depression, hope, and quality of life in dialysis patients using health promotion education groups. Hu Li Za Zhi. 2019;66(4):29–39. 31342499 10.6224/JN.201908_66(4).05

[27] Kaleta K, Mróz J. The relationship between basic hope and depression: forgiveness as a mediator. Psychiatr Q. 2020;91(3):877–86. 32361795 10.1007/s11126-020-09759-w PMC7395009

[28] Anczewska M, Wciórka J, Grygiel P, Nowak I, Sonik J, Świtaj P. Hope and its dimensions in relation to clinical recovery: a cross-sectional study among people with psychotic disorders. Psychiatr Rehabil J. 2019;42(2):139–46. 30556725 10.1037/prj0000340

[29] Oles SK, Fukui S, Rand KL, Salyers MP. The relationship between hope and patient activation in consumers with schizophrenia: results from longitudinal analyses. Psychiatry Res. 2015;228(3):272–6. 26165962 10.1016/j.psychres.2015.05.100

[30] Vrbova K, Prasko J, Ociskova M, Kamaradova D, Marackova M, Holubova M, et al. Quality of life, self-stigma, and hope in schizophrenia spectrum disorders: a cross-sectional study. Neuropsychiatr Dis Treat. 2017;13:567–76. 28260904 10.2147/NDT.S122483 PMC5328600

[31] Cook W, Kenny D. The actor-partner interdependence model: a model of bidirectional effects in developmental studies. Int J Behav Dev. 2005;29(2):101–9.

[32] Columbia Public Health. Dyadic Data Analysis. Columbia Public Health. 2019. Cited 2021 Aug 4. Available from: https://www-publichealth-columbia-edu.proxy.lib.duke.edu/research/population-health-methods/dyadic-data-analysis.

[33] Hsiao C-Y, Lu H-L, Tsai Y-F. Association between Mutuality and Health-related quality of life in patient-caregiver dyads living with Schizophrenia. Int J Environ Res Public Health. 2021;18(5):2438. 33801391 10.3390/ijerph18052438 PMC7967568

[34] Kay SR, Fiszbein A, Opler LA. The positive and negative syndrome scale (PANSS) for schizophrenia. Schizophr Bull. 1987;13(2):261–76. 3616518 10.1093/schbul/13.2.261

[35] World Health Organization. Process of translation and adaptation of instruments. World Health Organization. 2020. Cited 2022 Jan 28. Available from: https://www.who.int/substance_abuse/research_tools/translation/en/.

[36] Triveni D, Grover S, Chakrabarti S. Religiosity among patients with schizophrenia: an exploratory study. Indian J Psychiatry. 2017;59(4):420–8. 29497183 10.4103/psychiatry.IndianJPsychiatry_17_17 PMC5806320

[37] Ritsher JB, Otilingam PG, Grajales M. Internalized stigma of mental illness: psychometric properties of a new measure. Psychiatry Res. 2003;121(1):31–49. 14572622 10.1016/j.psychres.2003.08.008

[38] Stratton P, Lask J, Bland J, Nowotny E, Evans C, Singh R, et al. Detecting therapeutic improvement early in therapy: validation of the SCORE-15 index of family functioning and change. J Fam Ther. 2014;36(1):3–19.

[39] Stratton P, Bland J, Janes E, Lask J. Developing an indicator of family function and a practicable outcome measure for systemic family and couple therapy: the SCORE. J Fam Ther. 2010;32(3):232–58.

[40] Üsten et al. Measuring Health and Disability: Manual for WHO Disability Assessment Schedule (WHODAS 2.0). 2010;152. Cited 2022 Jan 27. Available from: https://apps.who.int/iris/handle/10665/43974.

[41] Reinhard SC, Gubman GD, Horwitz AV, Minsky S. Burden assessment scale for families of the seriously mentally ill. Eval Program Plann. 1994;17(3):261–9.

[42] Perry NS, Baucom KJW, Bourne S, Butner J, Crenshaw AO, Hogan JN, et al. Graphic methods for interpreting longitudinal dyadic patterns from repeated-measures actor-partner interdependence models. J Fam Psychol. 2017;31(5):592–603. 28240919 10.1037/fam0000293

[43] StatCorp. Stata statistical software. College Station, TX: StataCorp LLC; 2021.

[44] Vrbova K, Prasko J, Holubova M, Slepecky M, Ociskova M. Positive and negative symptoms in schizophrenia and their relation to depression, anxiety, hope, self-stigma and personality traits - a cross-sectional study. Neuro Endocrinol Lett. 2018;39(1):9–18. 29604619

[45] Olçun Z, Şahin AÖ. The correlation between schizophrenic patients’ level of internalized stigma and their level of hope. Arch Psychiatr Nurs. 2017;31(4):332–7. 28693867 10.1016/j.apnu.2017.03.001

[46] Lysaker PH, Salyers MP, Tsai J, Spurrier LY, Davis LW. Clinical and psychological correlates of two domains of hopelessness in schizophrenia. J Rehabil Res Dev. 2008;45(6):911–9. 19009477 10.1682/jrrd.2007.07.0108

[47] BelvederiMurri M, Amore M. The multiple dimensions of insight in Schizophrenia-Spectrum disorders. Schizophr Bull. 2019;45(2):277–83. 29939361 10.1093/schbul/sby092 PMC6403083

[48] Fortuna KL, Ferron J, Pratt SI, Muralidharan A, Aschbrenner KA, Williams AM, et al. Unmet needs of people with serious mental illness: perspectives from certified peer specialists. Psychiatr Q. 2019;90(3):579–86. 31154551 10.1007/s11126-019-09647-y

[49] Chan K, Wong FKY, Lee PH. A brief hope intervention to increase hope level and improve well-being in rehabilitating cancer patients: a feasibility test. SAGE Open Nurs. 2019;5:2377960819844381. 33415238 10.1177/2377960819844381 PMC7774404

[50] Laranjeira CA, Querido AIF. The multidimensional model of hope as a recovery-focused practice in mental health nursing. Rev Bras Enferm. 2022;75 Suppl 3(Suppl 3):e20210474. 10.1590/0034-7167-2021-0474 35239861

[51] Wamoyi J, Wight D, Remes P. The structural influence of family and parenting on young people’s sexual and reproductive health in rural northern Tanzania. Cult Health Sex. 2015;17(6):718–32. 25597368 10.1080/13691058.2014.992044 PMC4419469

[52] Iseselo MK, Kajula L, Yahya-Malima KI. The psychosocial problems of families caring for relatives with mental illnesses and their coping strategies: a qualitative urban based study in Dar es Salaam Tanzania. BMC Psychiatry. 2016;16:146. 27177934 10.1186/s12888-016-0857-y PMC4867081

[53] Magezi V. Changing family patterns from rural to urban and living in the in-between: a public practical theological responsive ministerial approach in Africa. HTS Teolog Stud. 2018;74(1):1–8.

