# Supplementary material for: Hopefulness among individuals living with schizophrenia and their caregivers in Tanzania: an actor-partner interdependence model
Source: BMC Psychiatry. 2023 Jul 13;23:508. doi: 10.1186/s12888-023-04990-8 (PMC10339619; doi:10.1186/s12888-023-04990-8)
Supplement: Supplementary file 1 — Additional file 1. Supplemental Table 1. HHI Item Scores by Time for PLWS & Caregivers. [file 12888_2023_4990_MOESM1_ESM.docx]

| **HHI Items**  *Mean (SD)* | **PLWS** | | **Caregivers** | |
| --- | --- | --- | --- | --- |
|  | *Baseline* | *Follow-Up* | *Baseline* | *Follow-Up* |
| 1. I have a positive outlook toward life. | 2.89 (0.11) | 3.33 (0.09) | 3.35 (0.09) | 3.57 (0.07) |
| 1. I have short and/or long-range goals. | 3.14 (0.10) | 3.43 (0.10) | 3.42 (0.08) | 3.62 (0.07) |
| 1. I feel all alone. | 2.52 (0.13) | 2.63 (0.13) | 3.00 (0.12) | 3.18 (0.11) |
| 1. I can see possibilities in the midst of difficulties. | 2.65 (0.09) | 2.92 (0.11) | 3.12 (0.08) | 3.40 (0.07) |
| 1. I have a faith that gives me comfort. | 3.08 (0.09) | 3.37 (0.09) | 3.37 (0.09) | 3.66 (0.07) |
| 1. I feel scared about my future. | 2.53 (0.13) | 2.48 (0.14) | 2.89 (0.13) | 2.68 (0.13) |
| 1. I can recall happy/joyful times. | 3.06 (0.08) | 3.37 (0.09) | 3.20 (0.09) | 3.43 (0.09) |
| 1. I have deep inner strength. | 2.62 (0.11) | 2.97 (0.10) | 3.09 (0.10) | 3.35 (0.09) |
| 1. I am able to give and receive caring/love. | 3.14 (0.10) | 3.37 (0.09) | 3.40 (0.10) | 3.71 (0.06) |
| 1. I have a sense of direction. | 2.45 (0.12) | 2.78 (0.12) | 3.14 (0.08) | 3.23 (0.10) |
| 1. I believe that each day has potential. | 2.95 (0.08) | 3.17 (0.10) | 3.31 (0.08) | 3.52 (0.07) |
| 1. I feel my life has value and worth. | 2.95 (0.11) | 3.49 (0.09) | 3.43 (0.09) | 3.65 (0.07) |

**Supplemental Table 1: HHI Item Scores by Time for PLWS & Caregivers.**

Items were scored using a four-point Likert scale ranging from 1 (strongly disagree) to 4 (strongly agree) and some items were reverse-coded prior to summing for the overall score.
